# Evaluation of a Pharmacist-Led Telephonic Medication Therapy Management Program in Rural Arizona: Implications for Community Health Practice

**DOI:** 10.3390/clinpract12030029

**Published:** 2022-04-22

**Authors:** David R. Axon, Jim Kloster, Becka Eckert, Sonia Morales, Sally Riggs, Aminata Kilungo, John Ehiri, Megan Grieser, Tenneh Turner-Warren, Teresa Aseret-Manygoats, Jennifer M. Bingham, Nicole Scovis, Terri Warholak

**Affiliations:** 1 R. Ken Coit College of Pharmacy, University of Arizona, Tucson, AZ 85721, USA; jkloster@pharmacy.arizona.edu (J.K.); beckert@pharmacy.arizona.edu (B.E.); smorales@pharmacy.arizona.edu (S.M.); mdgrieser@pharmacy.arizona.edu (M.G.); jbingham@trhc.com (J.M.B.); nscovis@trhc.com (N.S.); warholak@pharmacy.arizona.edu (T.W.); 2 Mel and Enid Zuckerman College of Public Health, University of Arizona Mel, Tucson, AZ 85724, USA; sriggs@arizona.edu (S.R.); paminata@arizona.edu (A.K.); jehiri@arizona.edu (J.E.); 3 Arizona Department of Health Services, Phoenix, AZ 85007, USA; tenneh.turner-warren@azdhs.gov (T.T.-W.); teresa.manygoats@azdhs.gov (T.A.-M.)

**Keywords:** medication therapy management, telehealth, rural health, pharmacist

## Abstract

This study evaluated a pharmacist-led telephonic Medication Therapy Management (MTM) program for rural patients in Arizona with poor access to healthcare services. A pharmacist provided telephonic MTM services to eligible adult patients living in rural Arizona communities with a diagnosis of diabetes and/or hypertension. Data were collected and summarized descriptively for demographic and health conditions, clinical values, and medication-related problems (MRPs) at the initial consultation, and follow-up data collected at 1 and 3 months. A total of 33 patients had baseline and one-month follow-up data, while 15 patients also had three-month follow-up data. At the initial consultation, the following MRPs were identified: medication adherence issues, dose-related concerns, adverse drug events (ADE), high-risk medications, and therapeutic duplications. Recommendations were made for patients to have the influenza, herpes zoster, and pneumonia vaccines; and to initiate a statin, angiotensin converting enzyme inhibitor, angiotensin receptor blocker, beta-blocker, and/or rescue inhaler. In conclusion, this study demonstrated that while pharmacists can identify and make clinical recommendations to patients, the value of these interventions is not fully realized due to recommendations not being implemented and difficulties with patient follow-up, which may have been due to the COVID-19 pandemic. Additional efforts to address these shortcomings are therefore required.

## 1. Introduction

Rural-dwelling patients, particularly low-income individuals, and older adults, often have considerable health disparities [1]. This is commonly because the demographic, economic, environmental, and social characteristics of individuals living in rural communities differ from those living in urban communities [2], and those living in rural areas have less access to healthcare facilities and healthcare professionals [3,4,5]. In addition, individuals living in rural communities are at greater risk of chronic diseases and a shorter life expectancy [2,6,7]. For example, a study looking at medical care and diabetes-related deaths from 2017 in Arizona found that those living in rural areas had a higher death rate compared to those living in an urban area [8]. Individuals living in rural communities typically have a greater prevalence of diabetes [3,9] and hypertension than those living in urban areas [10]. For instance, the Behavioral Risk Factor Surveillance System (BRFSS) estimated the prevalence of diabetes was 12.0% in rural areas and 10.4% in urban areas, while the prevalence of hypertension was 38.1% in rural areas and 32.6% in urban areas in 2013 [11]. Approximately 70% of patients with diabetes also have comorbid prehypertension or hypertension [3]. Managing diabetes is therefore one of the top three healthcare challenges for individuals living in rural communities, along with access to health care and nutrition and weight status [12]. One solution to help improve access to healthcare and subsequently improve health outcomes for rural-dwelling individuals is pharmacist-provided medication therapy management (MTM). MTM was established as part of the 2003 Prescription Drug, Improvement, and Modernization Act and includes several pharmacist-provided services [13,14]. These MTM services are offered to all eligible Medicare beneficiaries either in-person or via telephone and are designed to optimize health outcomes for patients [13,14,15]. Previous research has indicated the benefits of pharmacist delivered MTM services [16,17,18,19,20], although the scope, reach, and effectiveness of MTM services can sometimes be limited [21].

To address the unmet healthcare provision for individuals living in rural communities, a novel, interdisciplinary collaboration was established between a nationwide MTM service provider, a local community health center, the state health department, and an academic research team at the University of Arizona Colleges of Pharmacy and Public Health. The goal of this collaboration was to provide MTM services to individuals living in a rural Arizona community with diabetes and/or hypertension, to improve health outcomes. The objective of this study was to evaluate medication-related problems identified through a novel, collaborative pharmacist-provided MTM program for underserved patients living in a rural county in Arizona.

## 2. Methods

### 2.1. Project Design

This project was a collaboration between an academic research team at the University of Arizona Colleges of Pharmacy and Public Health, a university based MTM pharmacist, a Community Health Center, and the Arizona Department of Health Services. This program evaluation employed an interventional pre-post study design to evaluate this pharmacist-led MTM service for underserved patients in Arizona. 

### 2.2. Patient Eligibility

Any patient receiving healthcare services from a Community Health Center in rural Arizona were eligible for inclusion in this study if they were aged 18 years or older and diagnosed with type 2 diabetes mellitus and/or hypertension at the time of enrollment. Eligible patients were identified based on either a recent blood pressure greater than 140/90 mmHg or an A1c greater than 9%, as these indicated lack of disease state control.

### 2.3. MTM Service Provision

The community health center pharmacist performed a quality assessment to identify patients with the chronic conditions that met program criteria. These patients were referred to the university based MTM pharmacist via the electronic health record. The MTM pharmacist contacted eligible patients via telephone to offer them MTM services. The MTM pharmacist then provided telephonic MTM services to patients who consented to receive them, following the American Pharmacists Association’s Core Elements of an MTM Service Model [14]. The MTM service was primarily comprised of a comprehensive medication review (CMR), during which the pharmacist asked patients about all prescription and non-prescription medications prescribed or recommended by their providers. After the MTM service, the MTM pharmacist sent any relevant recommendations to the community health center pharmacist. Upon receiving the recommendations, the community health center pharmacist acted on the suggestions and followed up with healthcare providers at their facility. MTM services were provided to patients between July 2019 and June 2020.

### 2.4. Data Collection

Data were extracted from the patient’s health record into REDCap (Research Electronic Data Capture) Version 11.0.0 (Vanderbilt University, Nashville, TN, USA). REDCap is a secure, Health Insurance Portability and Accountability Act (HIPAA) complaint web-based platform designed to assist data capture for research studies [22,23].

Data were collected at the MTM consultation and again after 1 month and 3 months for: mean systolic and diastolic blood pressure, hemoglobin A1c, average fasting blood glucose, average postprandial blood glucose, medication adherence issues identified in the past 2 weeks (yes, no), gaps (i.e., not present when it was recommended) in vaccinations for influenza, herpes zoster, and pneumonia (where clinically indicated), and lack of adherence to national consensus guideline recommendations for the use of a statin, angiotensin converting enzyme inhibitor (ACEI) or angiotensin II receptor blocker (ARB), beta-blocker, rescue inhaler, and inhaled corticosteroid. Nonadherence to national consensus guideline recommendations were recorded dichotomously based on whether the patient should have had a prescription for a certain medication class (based on national consensus guidelines [24,25,26,27,28,29,30]) but did not. These medication classes were chosen because they represent treatments for prevalent chronic conditions and have clinical guidelines to support their use [24,25,26,27,28,29,30]. After a holistic assessment, if the MTM pharmacist determined that the patient had a lack of adherence to national consensus guideline recommendations, an electronic message within the electronic health record (EHR) was sent to the community health center pharmacist to inform them. Clinical lab tests were performed at the Community Health Center in rural Arizona. Data were reviewed by the pharmacist after 1 month and again after 3 months to determine if the pharmacists’ recommendations had been addressed. 

Data were also captured at baseline for medication-related problems (MRPs) and demographic and health characteristics. MRPs included: therapeutic duplications identified, drug-disease interactions identified, drug-drug interactions identified, dose-related concerns identified, adverse drug reactions identified, high-risk medications identified (e.g., Beer’s list of medications to avoid in the elderly) [31], need for hypoglycemia education, preventative screenings missing but recommended, and barriers to medication adherence. Barriers to medication adherence were selected based on the experience of the pharmacy team (pharmacists and technicians). Demographic and health characteristics collected included: age, gender, ethnicity, race, health insurance coverage, health conditions, patient health questionnaire-2 (PHQ-2) assessment score, and atherosclerotic cardiovascular disease 10-year risk score (where applicable). 

### 2.5. Data Analysis

Data were summarized using the appropriate statistics (i.e., frequencies with percentages for categorical data, and means with standard deviations for continuous data) using Microsoft Excel (version 16.53, Redmond, WA, USA). Due to the high amount of missing data, inferential statistics were not computed. The first analysis included all patients with baseline and 1-month follow-up data (*n* = 33). The secondary analysis included only those with baseline and 3-month follow-up data (*n* = 15). 

## 3. Results

The characteristics of study participants who had baseline characteristics and 1 month follow-up data (*n* = 33) are reported in Table 1. The mean age was 63.8 ± 13.9 years and the majority were female, Hispanic, and had health insurance provided by Medicaid. Of the 33 patients, 32 had diabetes and 30 had hypertension (these conditions were not mutually exclusive). Few patients had other health conditions including atherosclerotic cardiovascular disease (not myocardial infarction) (*n* = 1, 3.0%), heart failure (*n* = 1, 3.0%), asthma (*n* = 2, 6.1%), chronic obstructive pulmonary disease (*n* = 2, 6.1%), and atrial fibrillation (*n* = 2, 6.1%). Patient health questionnaire scores were almost exclusively ≤2, indicating no need for referral for most (97%) patients. Few patients had a therapeutic duplication, dose related concern, adverse drug event, or high-risk medication, while no patients had a drug–disease or drug–drug interaction. Hypoglycemia education was required for six (18.2%) patients. Several preventative screenings were required, most commonly including eye exams (*n* = 6), foot exams (*n* = 5), eye and foot exams (*n* = 5), and eye, foot, and renal exams (*n* = 3). Barriers to adherence included the medicine costing too much (*n* = 3), unable to get to the pharmacy (*n* = 1), and afraid of medicine reaction (*n* = 1), among others (*n* = 5). 

In the subgroup analysis sample of study participants who had baseline characteristics and 3-month follow-up data (*n* = 15; Table 1), the mean age was 61.4 ± 13.9 years and the majority were female, Hispanic, and had health insurance provided by Medicaid. All (*n* = 15) patients had diabetes and 14 had hypertension. Few patients had other health conditions including asthma (*n* = 2, 13.3%), chronic obstructive pulmonary disease (*n* = 2, 13.3%), and atrial fibrillation (*n* = 2, 13.3%). All patient health questionnaire scores were ≤2, indicating no need for referral. One patient had a therapeutic duplication, while no patients had a drug-disease or drug-drug interaction, dose related concern, adverse drug event, or high-risk medication. Hypoglycemia education was required for six patients. Several preventative screenings were required, most commonly including eye exams (*n* = 5), foot exams (*n* = 2), eye and foot exams (*n* = 2), and eye, foot, and renal exams (*n* = 2). Barriers to adherence included the medicine costing too much (*n* = 2), unable to get to the pharmacy (*n* = 1), and afraid of medicine reaction (*n* = 1), among others (*n* = 2).

Data for clinical lab values (e.g., systolic blood pressure, diastolic blood pressure, hemoglobin A1c, fasting blood glucose, and postprandial blood glucose) are summarized in Table 2 for the 1-month and 3-month follow-up data, but statistical differences were not calculated due to the large amount of missing data. 

Data on MRPs are also reported in Table 2. By the 1-month follow-up, the following had been addressed: two of the eight (25%) adherence issues, three of the ten (30%) flu vaccine recommendations, none of the twenty-five shingles vaccine recommendations, four of the 17 (23.5%) pneumonia vaccine recommendations, one of the seven (14.3%) statin initiation recommendations, one of the three (33.3%) ACEI/ARB initiation recommendations, none of the one beta-blocker initiation recommendations, and none of the one rescue inhaler initiation recommendations made.

In the subgroup analysis of 3-month follow-up data also reported in Table 2, the following had been addressed: one of the five (20%) adherence issues, three of the seven (42.9%) flu vaccine recommendations, none of the eleven shingles vaccine recommendations, and two of the eight (25%) pneumonia vaccine recommendations made.

## 4. Discussion

The main findings from this study were that pharmacist’s recommendations were not always acted upon, and that obtaining follow-up data was challenging. 

In this study, there were many instances where recommendations were made yet not acted upon at either the 1-month follow-up or the 3-month follow-up. For example, 10 patients had recommendations made for flu shots, yet only 3 of these patients had received their flu shot by the 1-month follow-up. Another study, although several years old now, identified that many individuals do not follow through on recommendations made to get vaccinated [32]. Acceptances for medications recommended in the current study ranged from 0% to 14%, although the numbers were low. A previous study of a telephonic MTM program found 37.5% of 200 pharmacist recommendations were accepted by providers [33]. This finding is somewhat similar to the findings in the current study. Although identifying medication or preventative health issues is an important first step, it has limited benefit if recommendations to address such issues are not acted upon. Many patients are unaware of the role that pharmacists play in medication therapy management, and far fewer understand the significance of this practice [34]. Greater efforts are therefore still required to improve awareness of MTM services and to increase the acceptance of pharmacists’ recommendations, to improve health outcomes. This finding also lends support to advocate for expanding the pharmacists’ scope of practice, or greater use of collaborative practice agreements. 

This study also identified issues with obtaining follow-up data. Missing data is often present in studies, and the extent of the missing data and the way it is dealt with can affect how the data may be interpreted [35]. While this is a common issue, it does limit the impact assessment of this program. At the end of the study, 3-month follow-up data were only available for 15 of the original 33 patients. This is lower than other studies reported in the literature. For example, a similar MTM program evaluation reported that approximately 70% of 1015 patients had follow-up data [36]. The current study may have been impacted by the COVID-19 pandemic that occurred during the program (1 July 2019, to June 2020). Although the MTM service was conducted telephonically, other in-person healthcare services may not have been as accessible. During the peak of the COVID-19 pandemic, when people were asked to stay at home wherever possible, it is likely that many patients did not attend follow-up in-person appointments or were unable to get lab tests conducted, and therefore it is logical that follow-up data are limited. For instance, one study outside the US reported up to a 40% decrease in follow-up visits [37]. Measurements such as blood pressure and HbA1c values were not able to be obtained since patients were not able to visit the clinic. 

Implications for policy and practice: The findings from this study offer insights to improve the facilitation of telephonic pharmacist-provided MTM service provision in rural areas. For instance, these results support the case for better integration with other health care team members to work collaboratively and help reduce loss to follow-up. To optimize the delivery of MTM services, it has been suggested that informal and formal relationships between health care professionals and pharmacists be fostered, and that health information be shared via EHRs [38]. For instance, a similar program found that the inclusion of nurses was important to the success of the program [33]. This approach could be employed here to help recruit and retain patients and help collect follow-up data for lab values and other data. It may be the case that using healthcare professionals who better represent the patient population being served could help improve service uptake and follow-up. For example, perhaps a Hispanic pharmacist would be more successful providing MTM services to Hispanic patients. Additional materials, such as written information about the MTM service, could be provided to patients to better inform them and retain them in the MTM service. Alternative working practices, such as the pharmacist adopting more flexible working hours to accommodate the patient’s schedule (e.g., evenings, weekends) or arranging a consultation in advance, may also help. Pharmacists may also be better integrated into the healthcare system, for instance, working in a healthcare clinic where they work collaboratively with other healthcare providers. This may help to facilitate a timely follow-up of the pharmacists’ recommendations with the physician or other healthcare provider. The findings of this study also support the calls for pharmacists to have provider status. This may help to make pharmacists recommendations to become reality, and capture follow-up data more effectively. It is important that policies and practices are revised to help improve access to MTM services for this population, given that a recent study estimated nonoptimized medication therapy related morbidity and mortality cost USD 528.4 billion in 2016; higher than the chronic conditions they were prescribed to treat, including heart disease (USD 230 billion) and diagnosed diabetes (USD 197 billion) [39]. 

There were limitations to this study beyond the amount of follow-up data. In particular, the small sample size of a narrowly defined local population (i.e., predominately Hispanic patients at one community health center in rural Arizona) limits the impact and external validity of the findings. Cultural differences among this population may have influenced the uptake of pharmacists’ recommendations. No written information about the program was provided to patients, which may also have limited the study. 

Future research should evaluate an expanded MTM program to gain additional data, perhaps from a broader population of eligible patients, and evaluate the impact of incorporating additional measures to increase outreach to eligible patients, e.g., offering greater flexibility in the pharmacists’ availability for MTM consultations. Additional efforts could also be made to access and obtain follow-up data, e.g., lab data from clinics, perhaps by employing an additional staff member to support the pharmacist. Future research should also assess a similar program in the post-COVID-19 era, to help eliminate the effects of the COVID-19 pandemic that likely influenced whether patients followed-up with their health providers and therefore the current study. It will also be appropriate to assess the economic implications or value of these services when larger sample sizes can be obtained. For instance, it would be interesting to assess if this program can reduce healthcare service utilization and all-cause healthcare expenditures. Finally, future work should investigate the barriers that exist to successfully implementing a telephonic, pharmacist provided MTM service for rural patients, which can inform the development or revision of MTM service provision for these underserved patients.

## 5. Conclusions

This study demonstrated that while pharmacists can identify and make clinical recommendations to patients, the value of these interventions is not fully realized due to recommendations not being implemented and difficulty following up with patients. Additional efforts to address these shortcomings are therefore required; for example, there is a need for healthcare providers to work closely with pharmacists to close this gap.

## Figures and Tables

**Table 1:** Demographic characteristics of patients with baseline and 1-month follow-up data (*n* = 33) and 3-month follow-up data (*n* = 15).

Variable	1-Month Follow-Up Data N (%) *	3-Month Follow-Up Data N (%) *
Age, mean ± SD	63.8 ± 13.9	61.4 ± 13.9
Female gender	24 (72.7)	11 (73.3)
Hispanic ethnicity	32 (97.0)	15 (100)
Health insurance coverage		
Medicaid	25 (75.8)	15 (100)
Medicare	16 (48.5)	5 (33.3)
Commercial	1 (3.0)	0 (0)
Health conditions		
Diabetes	32 (97.0)	15 (100)
Atherosclerotic Cardiovascular Disease (not Myocardial Infarction)	1 (3.0)	0 (0)
Heart failure	1 (3.0)	0 (0)
Hypertension	30 (90.9)	14 (93.3)
Asthma	2 (6.1)	2 (13.3)
Chronic Obstructive Pulmonary Disease	2 (6.1)	2 (13.3)
Atrial fibrillation	2 (6.1)	2 (13.3)
Patient Health Questionnaire-2 (PHQ-2) assessment score scored ≤2, no referral	32 (97.0)	15 (100)
Atherosclerotic Cardiovascular Disease 10-year risk (if applicable), mean ± SD (N = 20)	22.4 ± 21.5	20 ± 21.3
Therapeutic duplications identified	1 (3.0)	1 (6.7)
Drug-disease interactions identified	0 (0)	0 (0)
Drug-drug interactions identified	0 (0)	0 (0)
Dose related concerns identified	2 (6.1)	0 (0)
Adverse drug reactions identified	1 (3.0)	0 (0)
High-risk medications identified	1 (3.0)	0 (0)
Need for hypoglycemia education	6 (18.2)	6 (40.0)
Preventative screenings missing, but recommended		
None	11 (33.3)	3 (20.0)
Eye exam	6 (18.2)	5 (33.3)
Eye and foot exam	5 (15.2)	2 (13.3)
Eye, foot, renal exam	3 (9.1)	2 (13.3)
Foot exam	5 (15.2)	2 (13.3)
Renal exam	1 (3.0)	0 (0)
Dental exam	1 (3.0)	0 (0)
Missing	1 (3.0)	1 (6.7)
Barriers to adherence		
Patient thought medicine cost too much	3 (9.1)	2 (13.3)
Patient couldn’t get to the pharmacy	1 (3.0)	1 (6.7)
Patient was afraid of medicine reactions	1 (3.0)	1 (6.7)
Other	5 (15.2)	2 (13.3)

SD = standard deviation; * = unless otherwise indicated. Note that health insurance coverage and health conditions were not mutually exclusive categories.

**Table 2:** Variables with baseline and 1-month follow-up data (N = 33) and 3-month follow-up data (N = 15).

Variable	1-Month Follow-Up Data N (%) *	3-Month Follow-Up Data N (%) *
Baseline systolic blood pressure, mean ± SD	N = 33: 133.5 ± 20.4	N = 15: 132.8 ± 19.4
Follow-up systolic blood pressure, mean ± SD	N = 10: 141.5 ± 31.2	N = 6: 133.7 ± 20.1
Baseline diastolic blood pressure, mean ± SD	N = 33: 78.5 ± 10.4	N = 15: 80.7 ± 9.5
Follow-up diastolic blood pressure, mean ± SD	N = 10: 81 ± 16.3	N = 6: 78.3 ± 5.1
Baseline hemoglobin A1c, mean ± SD	N = 32: 9.9 ± 2.3	N = 15: 10.8 ± 2.0
Follow-up hemoglobin A1c, mean ± SD	N = 10: 10.1 ± 1.7	N = 7: 8.0 ± 1.8
Baseline average fasting blood glucose, mean ± SD	N = 22: 167.5 ± 70.4	N = 10: 161.9 ± 73.0
Follow-up average fasting blood glucose, mean ± SD	N = 4: 129.3 ± 35.6	N = 1: 140 ± 0
Baseline average postprandial blood glucose, mean ± SD	N = 2: 212.5 ± 123.7	N = 2: 212.5 ± 123.7
Follow-up average postprandial blood glucose, mean ± SD (N = 0)	-	-
Adherence issue identified	8 (24.2)	5 (33.3)
Addressed by follow-up	2 (25.0)	1 (20.0)
Flu vaccine missing and recommended	10 (30.3)	7 (46.7)
Addressed by follow-up	3 (30.0)	3 (42.9)
Shingles vaccine missing and recommended	25 (75.8)	11 (73.3)
Addressed by follow-up	0 (0)	0 (0)
Pneumonia vaccine missing and recommended	17 (51.5)	8 (53.3)
Addressed by follow-up	4 (23.5)	2 (25.0)
Statin missing and recommended	7 (21.2)	
Addressed by follow-up	1 (14.3)	
Angiotensin converting enzyme inhibitor/angiotensin II receptor blocker missing and recommended	3 (9.1)	
Addressed by follow-up	1 (33.3)	
Beta-blocker missing and recommended	1 (3.0)	
Addressed by follow-up	0 (0)	
Rescue inhaler missing and recommended	1 (3.0)	
Addressed by follow-up	0 (0)	
Inhaled corticosteroid missing and recommended	0 (0)	
Addressed by follow-up	0 (0)	

SD = standard deviation; * = unless otherwise indicated. Note that medications were not followed-up at the 3-month follow-up.

## Data Availability

De-identified data are available upon request from the corresponding author.
